# Correlation analysis between the prevalence of common respiratory pathogens and exposure to ambient air pollutants in Central China, 2014–2022

**DOI:** 10.3389/fpubh.2025.1532507

**Published:** 2025-03-06

**Authors:** Yaqi Zhu, Yuying Hou, Ting Xiang, Yingtao Wu, Xiaojian Cao, Xiaoxue Wu, Jinya Ding, Xionghui Zhou, Xiaohua Chen

**Affiliations:** ^1^Department of Laboratory Medicine, General Hospital of Central Theater Command, Wuhan, China; ^2^Department of Laboratory Medicine, Maternal and Child Health Hospital of Hubei Province, Wuhan, China; ^3^Hubei Key Laboratory of Agricultural Bioinformatics, College of Informatics, Huazhong Agricultural University, Wuhan, China; ^4^Department of Rehabilitation Medicine, General Hospital of Central Theater Command, Wuhan, China; ^5^State Key Laboratory of Agricultural Microbiology, College of Veterinary Medicine, Huazhong Agricultural University, Wuhan, China; ^6^Department of Clinical Laboratory, Maternal and Child Health Hospital of Hubei Province, Wuhan, China; ^7^The First School of Clinical Medicine, Southern Medical University, Guangzhou, China

**Keywords:** respiratory pathogens, respiratory tract infections, epidemiological characteristics, air pollutants, prevalence

## Abstract

**Background:**

Whether ambient air pollutants affect the transmission of respiratory pathogens in central Wuhan is unknown. So, we conducted a series of statistical analyses to discover the correlation between the two.

**Methods:**

We enrolled a total of 47,668 outpatient and hospitalized patients who underwent IgM antibody tests for nine types of respiratory pathogens, namely, *Legionella pneumophila* type 1 (LP1), *Mycoplasma pneumoniae* (MP), Q fever rickettsia (QFR), *Chlamydia pneumoniae* (CP), adenovirus (ADV), respiratory syncytial virus (RSV), influenza virus A (FluA), influenza virus B (FluB), and parainfluenza virus (PIVs) between January 2014 and December 2022. Monthly measurements were taken for specific air pollutants, including fine particulate matter 2.5 (PM2.5), inhalable particulate matter 10 (PM10), ozone (O_3_), nitrogen dioxide (NO_2_), sulfur dioxide (SO_2_), and carbon monoxide (CO) at the same periods. The association between different respiratory pathogen infections and major air pollutants was primarily analyzed using Spearman’s correlation analysis.

**Conclusion:**

MP, LP1, and FluB are correlated with respiratory infections and have been identified as potential causative agents. Elevated levels of O_3_ were found to augment the incidence of MP infection. We first discovered the positive correlation between SO_2_ and ADV infection and between CO and LP1 infection. The presence of air pollutants in Wuhan showed a significant correlation with respiratory pathogens, and elevated levels of air pollution facilitated their transmission to individuals.

## Introduction

Respiratory tract infections (RTIs) pose a global disease burden, with an estimated annual mortality rate exceeding 4 million deaths (1). Mild symptoms typically include fever, coughing episodes accompanied by chest pain, and breathing difficulties; however, severe cases progress into dyspnea or hypoxia, eventually resulting in respiratory failure or even death (2, 3). An estimated 544.9 million individuals worldwide were affected by chronic respiratory diseases in 2017, representing a 39.8% increase from 1990 (4). Respiratory pathogens, such as *Legionella pneumophila* type 1 (LP1), *Mycoplasma pneumoniae* (MP), Q fever rickettsia (QFR), *Chlamydia pneumoniae* (CP), adenovirus (ADV), respiratory syncytial virus (RSV), influenza virus A (FluA), influenza virus B (FluB), and parainfluenza virus (PIVs), are the primary causative agents responsible for RTIs in infants, children, and adults.

There is an association between severe air pollution and an elevated risk of respiratory pathogen infections. Numerous epidemiological studies have consistently shown that short-term fluctuations in exposure to ambient air pollution are linked to adverse health outcomes (5–7). Atmospheric particulate matter (PM) can adsorb a multitude of pathogenic microorganisms or various harmful substances, infiltrating the human respiratory tract by mediating inflammatory responses and oxidative stress and penetrating deep into lung tissue (8, 9). Exposure to air pollutants has been shown to induce oxidative stress, leading to free radical production, which could potentially impair the respiratory system and reduce resistance against bacterial and viral infections (10). Huang et al. have pointed out the effects of some air pollutants, such as PM2.5, PM10, and NO_2_, on respiratory tract infections in Nanjing (China) (11). A study conducted in America revealed that exposure to a certain concentration of O_3_ can induce the expression of inflammatory markers, trigger an inflammatory response, and enhance susceptibility to influenza viruses (12). Previous investigations have also established a positive correlation between air pollutant concentrations and the incidence of respiratory infections among children, indicating that inhalation of air pollutants can disrupt the micro-ecology of the respiratory tract and increase the susceptibility to respiratory infections (13). Over the past two decades, Ciencewicki et al. reported an insightful review regarding the potential interactions between various air pollutants and respiratory virus infections. This comprehensive study highlighted how exposure to these common air pollutants (NO_2_, O_3_, and PM) modulated the host immunity against respiratory virus infections, meaning some profound implications for global public health (10).

Despite previous studies examining the correlation between specific air pollutants and some respiratory pathogens, there remains a lack of research emphasizing the relationship between six common air pollutants and nine distinct respiratory pathogens. First, our study provides a comprehensive analysis of six air pollutants and nine respiratory pathogens, including viruses, mycoplasma, chlamydia, and rickettsia. Second, this research covers an extensive time span and includes participants from a wide age distribution, rendering it highly representative of the region. Third, we revealed the susceptibility of distinct age groups to specific respiratory pathogens under different air pollutants. Finally, we explored the impact of high- and low-concentration groups of air pollutants on the prevalence of respiratory pathogens for the first time at home and abroad. We revealed that high concentrations of air pollutants can significantly increase the prevalence of certain respiratory pathogens.

## Materials and methods

### Data collection

The study comprised respiratory pathogen sample testing data from the General Hospital of Central Theater Command from 2014 to 2022. The comprehensive datasets included nearly 48,000 samples, with each individual sample meticulously examined for the presence of nine respiratory pathogens (LP1, MP, QFR, CP, ADV, RSV, FluA, FluB, and PIVs) using IFA of IgM antibody tests with PNEUMOSLIDE IgM kit (VIRCELL, S.L. Granada, Espańa). The relative prevalence of each pathogen is defined as dividing its corresponding positive count by the total number of positives. In addition, we noted that these subjects were all tested for nine respiratory pathogens when they came to the hospital with symptoms of respiratory infection.

This study incorporated two sets of air pollution index data from different sources. The first set encompassed the period between 2014 and 2019, sourced from the National Urban Air Quality Real-time Publishing Platform of China Environmental Monitoring General Station. The second set covered the period from 2020 to 2022, obtained from Hubei Province’s Department of Ecology and Environment. Specifically, data pertaining to the Wuhan region were selected for this research. Except for O_3_, which is measured as the daily maximum 8-h average concentration, PM2.5, PM10, SO_2_, NO_2_, and CO are all assessed based on their 24-h average concentrations. The concentrations of six air pollutants are represented by the mean with their 95% confidence intervals (95% *CI*). A *p*-value of <0.05 indicates statistical significance.

### Statistical analysis

Python 3.8.17 was employed for data entry and analysis. For the air pollutant data, we calculated average values of the concentration and 95% confidence intervals (95% *CI*) for all six pollutants annually. The “chi2_contingency” function from the “scipy.stats” library 1.11.4 was utilized to conduct chi-square tests on the pollutant indicators. The yearly status of the six air pollutants is presented in Tables 1, 2. Regarding the respiratory pathogen sample testing data, we computed the positive rates for each pathogen over a 9-year period. Simultaneously, we classified the samples into five age groups based on human growth and development stages: <1 year, 1–7 years, 7–18 years, 18–65 years, and > 65 years. The positive rates of each respiratory pathogen in different age groups are presented in Table 3. To investigate the relationship between air pollutant levels and respiratory pathogen positivity, we performed a correlation analysis using the “spearmanr” function from the “scipy.stats” library. For air pollutants and respiratory pathogens exhibiting strong associations, we further conducted a least-squares linear regression analysis; the “OLS” function from the “statsmodels” library is used. In addition, we divided the concentration of air pollutants into a high-concentration group (top 35%) and a low-concentration group (bottom 35%) to determine whether the high-concentration group can increase the prevalence of respiratory pathogens. Subsequently, Mann–Whitney *U*-tests were employed to assess differences in positive rates between these two groups, which uses the “mannwhitneyu” function from the “scipy.stats” library. A two-tailed hypothesis test is employed to assess the statistical significance of the observed effect.

**Table 1:** Concentration of air pollutants, 2014–2019.

	Air Pollutants							
Year	PM2.5 (μg/m^3^) [95% CI]	PM10 (μg/m^3^) [95% CI]	O_3_ (μg/m^3^) [95% CI]	NO_2_ (μg/m^3^) [95% CI]	SO_2_ (μg/m^3^) [95% CI]	CO (mg/m^3^) [95% CI]	χ^2^	*P*
2014	177.15 [159.89–194.41]	96.77 [84.67–108.87]	60.31 [47.63–73.00]	35.59 [32.57–38.61]	22.04 [18.39–25.69]	13.81 [12.05–15.56]	162.78	1.35e-12
2015	164.69 [149.52–179.87]	92.30 [81.88–102.72]	61.79 [52.28–71.31]	34.16 [30.75–37.57]	19.02 [15.98–22.06]	16.95 [14.47–19.42]	123.17	3.91e-07
2016	143.76 [126.16–161.37]	79.87 [71.28–88.47]	53.23 [42.73–63.73]	30.68 [28.23–33.12]	8.85 [6.02–11.67]	12.45 [10.91–14.00]	134.44	1.34e-08
2017	112.15 [85.79–138.51]	61.55 [44.62–78.47]	36.65 [26.56–46.74]	26.34 [19.42–33.25]	5.00 [3.42–6.57]	10.50 [8.26–12.73]	111.25	1.49e-06
2018	132.49 [119.26–145.72]	65.87 [58.12–73.63]	48.17 [38.21–58.13]	28.10 [25.58–30.61]	6.40 [5.61–7.19]	12.02 [11.18–12.85]	145.24	4.50e-10
2019	129.71 [116.93–142.49]	62.60 [56.07–69.12]	49.77 [36.20–63.34]	26.61 [23.83–29.38]	5.28 [4.46–6.10]	11.19 [10.13–12.25]	198.06	4.95e-18

**Table 2:** Concentration of air pollutants, 2020–2022.

	Air pollutants							
Year	PM2.5 (μg/m^3^) [95% CI]	PM10 (μg/m^3^) [95% CI]	O_3_ (μg/m^3^) [95% CI]	NO_2_ (μg/m^3^) [95% CI]	SO_2_ (μg/m^3^) [95% CI]	CO (mg/m^3^) [95% CI]	χ^2^	*P*
2020	37.33 [28.01–46.64]	57.39 [46.39–68.40]	92.50 [76.56–108.44]	36.02 [28.86–43.17]	7.92 [6.95–8.89]	0.85 [0.80–0.91]	244.08	1.20e-25
2021	36.08 [27.11–45.05]	56.64 [46.05–67.22]	95.16 [78.21–112.11]	40.18 [33.63–46.72]	7.78 [6.95–8.62]	0.83 [0.77–0.88]	258.69	3.69e-28
2022	34.61 [23.81–45.41]	54.27 [44.94–63.59]	100.86 [82.91–118.81]	34.37 [29.06–39.68]	8.52 [7.88–9.17]	0.86 [0.80–0.91]	289.78	1.29e-33

**Table 3:** Sample numbers and positive proportions in different age groups, 2014–2022.

			Age groups					
Year			<1 year No.(%)	1–7 year No.(%)	7–18 year No.(%)	18–65 year No.(%)	>65 year No.(%)	Total No.(%)
2014-2022	Samples received		4,607(9.66)	20,924(43.90)	4,141(8.69)	11,001(23.08)	6,995(14.67)	47,668
	Gender, No. (%)	Male	2,917(6.12)	12,136(25.46)	2,466(5.17)	7,377(15.48)	4,805(10.08)	29,701(62.31)
		Female	1,690(3.54)	8,788(18.44)	1,675(3.52)	3,624(7.60)	2,190(4.59)	17,967(37.69)
	LP1		20(0.43)	848(4.05)	322(7.78)	709(6.44)	270(3.86)	2,169(4.55)
	MP		289(6.27)	6,430(30.73)	1,056(25.50)	1,531(13.92)	408(5.83)	9,714(20.38)
	QRF		1(0.02)	104(0.5)	23(0.56)	55(0.50)	12(0.17)	195(0.41)
	CP		1(0.02)	88(0.42)	108(2.61)	238(2.16)	23(0.33)	458(0.96)
	ADV		13(0.28)	104(0.5)	14(0.34)	22(0.20)	13(0.19)	166(0.35)
	RSV		65(1.41)	38(0.18)	6(0.14)	32(0.29)	20(0.29)	161(0.34)
	FluA		3(0.07)	19(0.09)	1(0.02)	5(0.05)	7(0.10)	35(0.07)
	FluB		23(0.50)	938(4.48)	123(2.97)	114(1.04)	65(0.93)	1,263(2.65)
	PIVs		17(0.37)	410(1.96)	61(1.47)	91(0.83)	47(0.67)	626(1.31)

To assess the association between various air pollutants and respiratory pathogen infection, we initially computed the *p*-value. A *p*-value of <0.05 or a -Log10 (*p-*value) > 1.3 was considered a statistically significant difference. Spearman’s correlation analysis was subsequently employed to determine Spearman’s *ρ*, which evaluates whether a significant relationship exists between air pollutants and respiratory pathogen infections. When Spearman’s ρ > 0, it indicates a positive correlation between the two variables. As the value of this coefficient increases, so does the significance of this positive correlation.

### Ethics statement

This study was approved by the Ethics Committee of the General Hospital of Central Theater Command as the lead center. The Ethics Committee of the General Hospital of Central Theater Command waived the requirement for informed consent due to the retrospective nature of the study. All methods were performed in accordance with the relevant guidelines and regulations.

## Results

### Distribution and positive rates of respiratory pathogens in outpatient and hospitalized patients, 2014–2022

**Figure 1:**
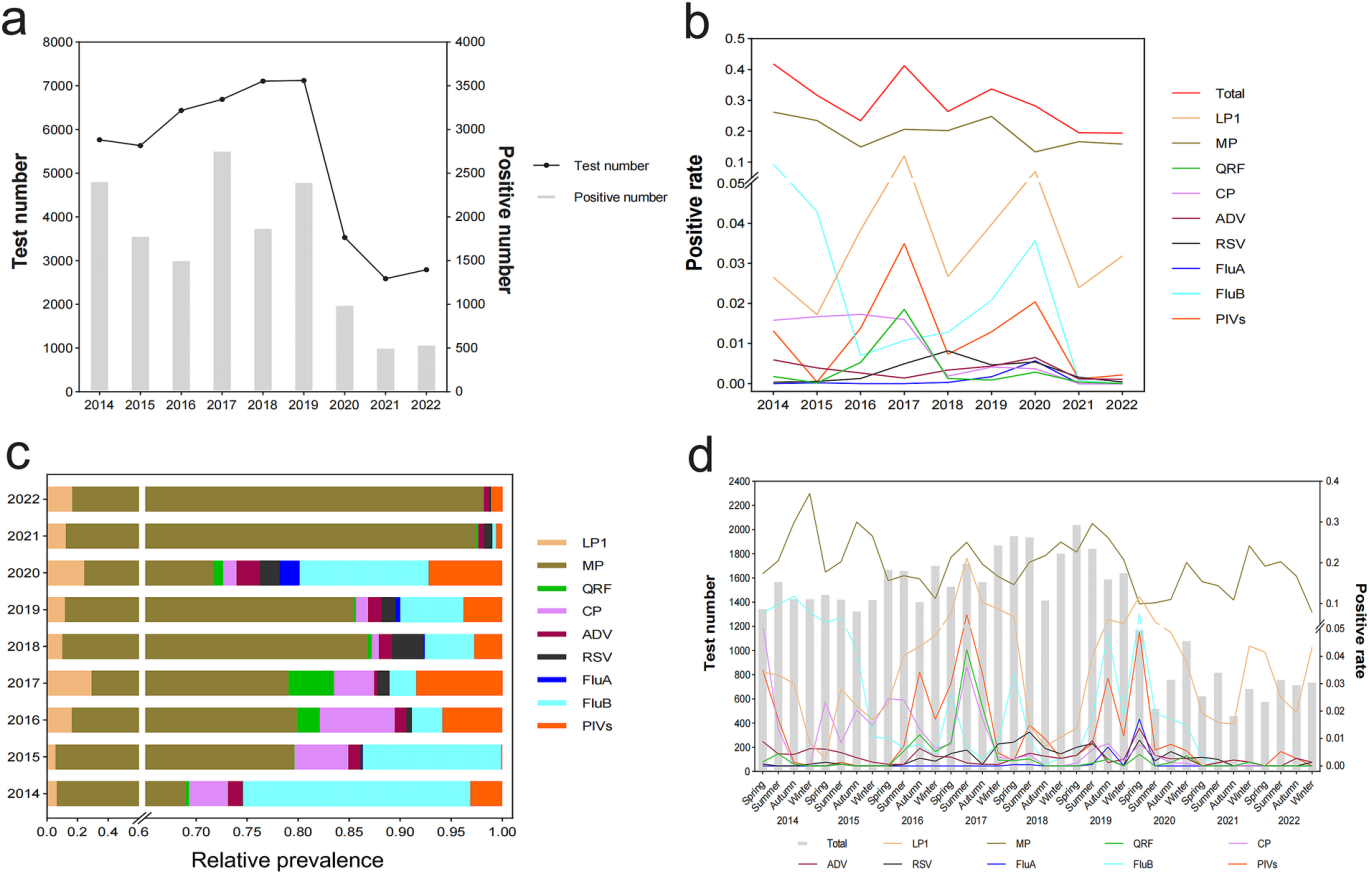
Distribution and detection rates of nine respiratory pathogens in 47,668 outpatient and hospitalized patients, January 2014 to December 2022. Patients were tested for LP1, MP, QRF, CP, ADV, RSV, FluA, FluB, and PIVs using a PNEUMOSLIDE IgM kit. **(A)** Total number of tests and number of positive tests by year. **(B)** Overall positive rate and positivity rates by pathogens type and year. **(C)** Relative prevalence of each respiratory pathogen by year. **(D)** Quarterly distribution and detection rates of nine respiratory pathogens. LP1, *Legionella pneumophila* type 1; MP, *Mycoplasma pneumoniae*; QRF, Q fever rickettsia; CP, *Chlamydia pneumoniae*; ADV, adenovirus; RSV, respiratory syncytial virus; FluA, influenza A; FluB, influenza B; PIVs, parainfluenza virus.

The distribution of the total and positive number of tests for nine respiratory pathogens was calculated annually and analyzed by grouping from 2014 to 2022. We revealed that the total number of people tested increased yearly from 2015 to 2019, with the highest number of tests conducted in 2019 (7,120 cases). However, after the outbreak of COVID-19 in Wuhan in 2020, the number of tests sharply plummeted and reached its lowest point in 2021 (2,589 cases). Notably, the positive number of cases fluctuated during this 9-year period. Specifically, it peaked in 2017 (2,761 cases) and decreased to its lowest level in 2021 (505 cases) (Figure 1A). The total positive rates of respiratory pathogens reached their highest levels in 2014 (41.8%) and 2017 (41.3%). Notably, MP consistently exhibited the highest positive rate each year. The remaining eight pathogens presented varying degrees of fluctuations. The prevalence of QFR, RSV, and FluA experienced its peak in 2017, 2018, and 2020, respectively. FluB emerged as the second most prevalent pathogen after MP from 2014 to 2015. LP1 and PIVs became highly dominant pathogens in addition to MP in 2017. However, LP1, PIVs, and QFR demonstrated a significant declining trend inversely by 2018. Except for MP and LP1, the prevalence rates of the other seven pathogens approached zero from 2021 to 2022 (Figure 1B). MP consistently exhibits the highest relative prevalence and becomes the primary causative agent for respiratory infections, particularly in 2021 and 2022. However, the relative prevalence of other pathogens achieved their zenith observed in the following years, specifically LP1 (2017, 29.1%), FluB (2014, 22.3%), PIVs (2017, 8.5%), CP (2016, 7.4%), QRF (2017, 4.5%), RSV (2018, 3.1%), ADV (2020, 2.3%), and FluA (2020, 2.0%) (Figure 1C). Each pathogen exhibits their own distinct characteristics of the epidemic. The prevalence of MP, which can occur throughout the study period, is exceedingly higher than other pathogens and easier to be prevalent during autumn and winter. LP1 is the second most prevalent pathogen in terms of popularity and is more common in summer and spring at its peak. In addition, FluA shows sporadic occurrences with a relatively lower incidence rate during spring and summer. RSV and ADV have consistently maintained a remarkably low prevalence, and QFR infection was also negligible. The prevalence of other pathogens remained at a moderate level over the 9-year period. PIVs can be seen most in the spring and autumn seasons. FluB is most prevailing in spring and autumn, while CP is in spring and summer. After the COVID-19 outbreak in 2021 and 2022, the infection rates of the other seven respiratory pathogens decreased almost to 0 except for MP and LP1 (Figure 1D).

### Difference and correlation analysis between air pollutants and respiratory pathogen infections

**Figure 2:**
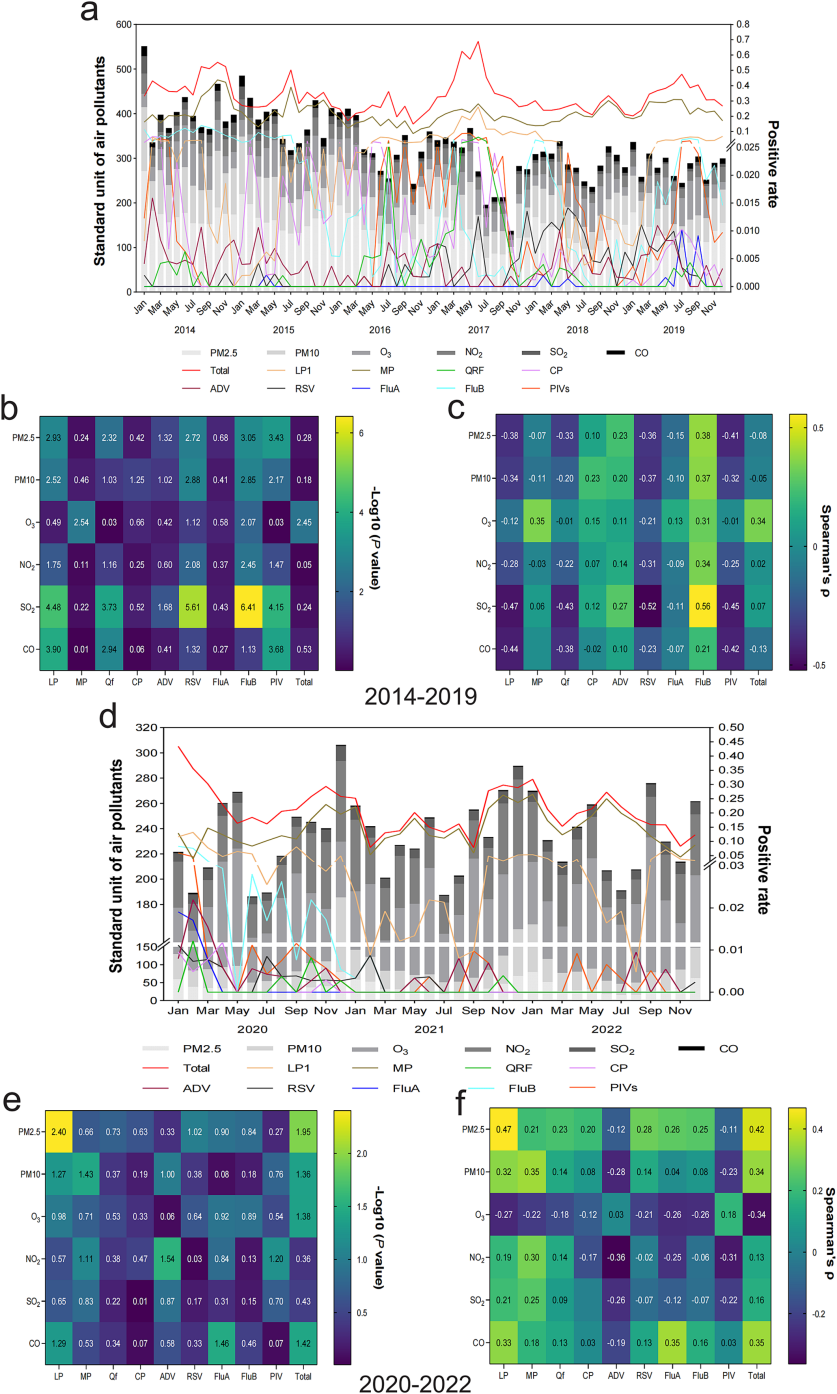
Relationship between air pollutants and the prevalence of respiratory pathogens. **(A)** An overview of the composition of six types of air pollutants combined with the prevalence of nine respiratory pathogens every month, 2014–2019. **(B)** Differences between various air pollutants and respiratory pathogens represented by -Log10 (*p-*value), 2014–2019. A -Log10 (*p-*value) > 1.3 was considered a statistically significant difference. **(C)** Association between different air pollutants and respiratory pathogens represented by Spearman’s rank correlation coefficient (Spearman’s *ρ*), 2014–2019. Spearman’s *ρ* ranges from −1 to 1, with positive values suggesting a positive correlation and negative values indicating a negative correlation. The larger the absolute value, the more significant the correlation. Spearman’s *ρ* value is reported with a precision of two decimal places, and an empty entry indicates a value less than 0.01. **(D)** An overview of the composition of six types of air pollutants combined with the prevalence of nine respiratory pathogens every month, 2020–2022. **(E)** Differences between various air pollutants and respiratory pathogens represented by -Log10 (*p-*value), 2020–2022. **(F)** Association between different air pollutants and respiratory pathogens represented by Spearman’s *ρ*, 2020–2022.

A simple addition of six air pollutant concentrations can partially reflect the overall degree of air pollution in a given month. However, the positive rate of specific respiratory pathogens is related to some air pollutants rather than considering a direct association between overall air pollution levels and the incidence of respiratory pathogens. An overview of air pollution with respiratory infection from 2014 to 2019 is shown in Figure 2A. There is a relationship between air pollutant concentrations and respiratory pathogen infections. Specifically, MP is greatly influenced by the concentration of O_3_. When it increased, the positive rate of MP also tended to be relatively high. Similarly, higher SO_2_ concentrations correspondingly led to an increased ADV positive rate. FluB is the most susceptible to the influence of air pollutants compared to other pathogens. Higher concentrations of PM2.5, PM10, O_3_, NO_2_, and SO_2_ could increase its positive rate, except for CO, which does not have an obvious impact. The result of difference and correlation analysis between air pollutants and respiratory infections is revealed in Figures 2B,C, respectively; the combinations that conform to both conditions of significant difference and positive correlation were FluB and SO_2_, FluB and NO_2_, FluB and O_3_, FluB and PM2.5, FluB and PM10, ADV and SO_2_, and MP and O_3_. Inversely, SO_2_ was negatively associated with RSV and LP1. The relationship between air pollution and respiratory infection in 2020–2022 has undergone significant changes compared to 2014–2019 (Figure 2D). Specifically, an increase in PM10 levels is associated with a corresponding rise in MP infection. Similarly, concentrations of CO are positively associated with the prevalence of LP1. Conversely, SO_2_ levels have remained relatively stable during this 3-year period and have had little impact on the incidence of respiratory pathogens. Particularly, seasonal epidemics of FluA, FluB, CP, and QRF only occurred in 2020. Their prevalence decreased to 0 over the following 2 years without any discernible associations with air pollution. Figures 2E,F demonstrate the significant positive correlation between respiratory infection and air pollutants, which were MP and PM10, as well as LP1 and CO, while a negative correlation between NO_2_ and ADV during epidemic periods was observed.

### Linear correlation between air pollutants and respiratory pathogen infections with regression analysis

**Figure 3:**
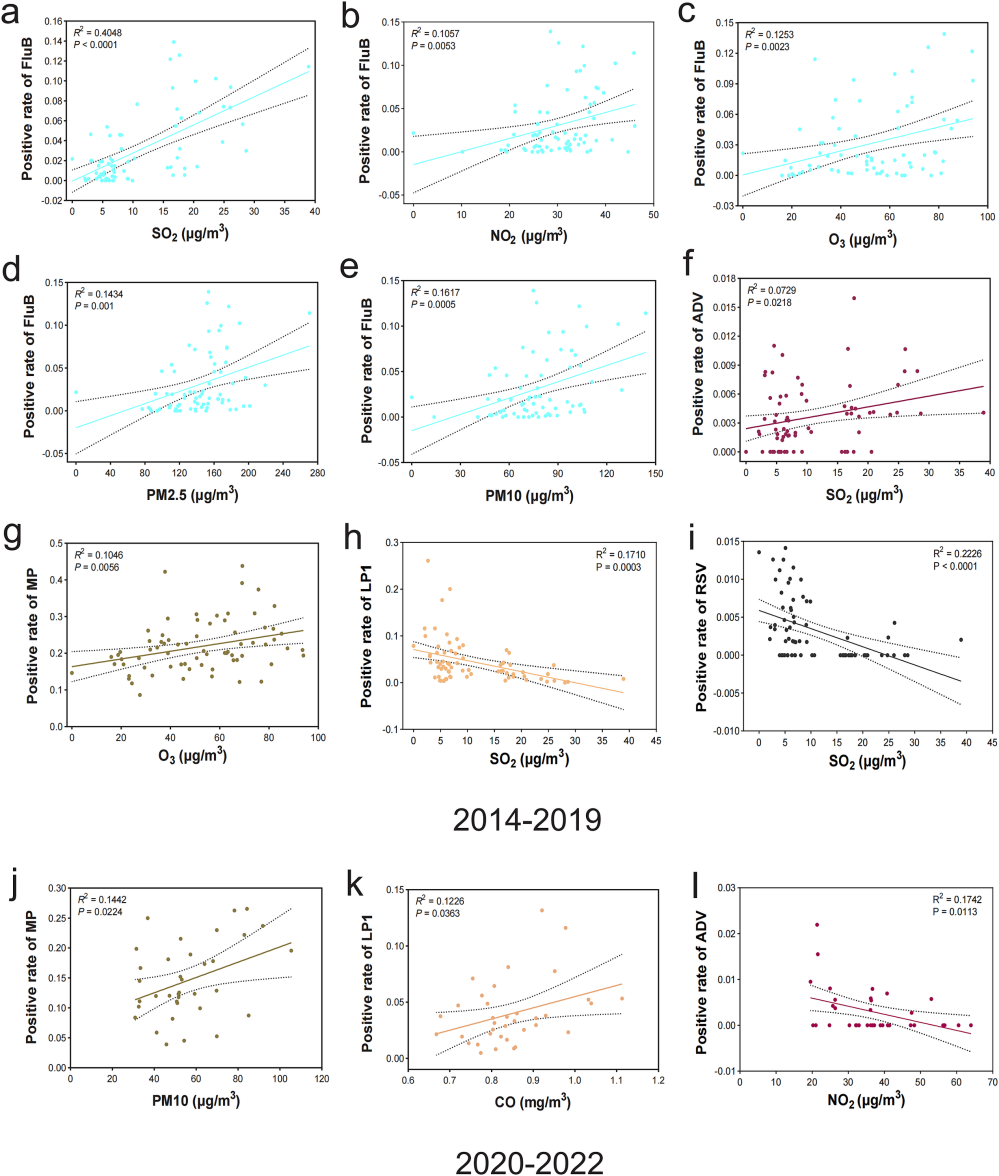
Correlation analysis of air pollutants and respiratory pathogens. **(A–G)** Positive correlation between the prevalence of FluB and SO_2_ (*p <* 0.0001), FluB and NO_2_ (*p =* 0.0053), FluB and O_3_ (*p =* 0.0023), FluB and PM2.5 (*p =* 0.001), FluB and PM10 (*p =* 0.0005), ADV and SO_2_ (*p =* 0.0218), and MP and O_3_ (*p =* 0.0056), 2014–2019. **(H,I)** Negative correlation between the prevalence of LP1 and SO_2_ (*p =* 0.0003) and RSV and SO_2_ (*p <* 0.0001), 2014–2019. **(J,K)** Positive correlation between the prevalence of MP and PM10 (*p =* 0.0224) and LP1 and CO (*p =* 0.0363), 2020–2022. **(L)** Negative correlation between the prevalence of ADV and NO_2_ (*p =* 0.0113), 2020–2022. Statistical significance was set at a *p*-value of < 0.05.

A regression analysis was conducted to comprehensively examine the significant linear correlation between air pollutants and respiratory pathogens and quantify the level of concordance between these two variables. The results of period in 2014–2019 demonstrated that there were significantly positive linear correlation among the following groups: FluB and SO_2_ (*r*^2^ = 0.4048, *p <* 0.0001) (Figure 3A), FluB and NO_2_ (*r*^2^ = 0.1057, *p* = 0.0053) (Figure 3B), FluB and O_3_ (*r*^2^ = 0.1253, *p* = 0.0023) (Figure 3C), FluB and PM2.5 (*r*^2^ = 0.1434, *p* = 0.001) (Figure 3D), FluB and PM10 (*r*^2^ = 0.1617, *p* = 0.0005) (Figure 3E), ADV and SO_2_ (*r*^2^ = 0.0729, *p* = 0.0218) (Figure 3F), MP and O_3_ (*r*^2^ = 0.1046, *p* = 0.0056) (Figure 3G), while showed a characteristic negative correlation were LP1 and SO_2_ (*r*^2^ = 0.1710, *p* = 0.0003) (Figure 3H), RSV and SO_2_ (*r*^2^ = 0.2226, *p <* 0.0001) (Figure 3I). In the period of 2020–2022, MP and PM10 (*r*^2^ = 0.1442, *p* = 0.0224) (Figure 3J), LP1 and CO (*r*^2^ = 0.1226, *p* = 0.0363) (Figure 3K) were presented positive linear correlation. However, ADV and NO_2_ (*r*^2^ = 0.1742, *p* = 0.0113) (Figure 3L) manifested a negative correlation. Correlations between air pollutants and respiratory pathogen infections are summarized in Table 4.

**Table 4:** Correlation between air pollutants and respiratory pathogens infections, 2014–2022.

	Air pollutants					
Pathogens	PM2.5 (μg/m^3^)	PM10 (μg/m^3^)	O_3_ (μg/m^3^)	NO_2_ (μg/m^3^)	SO_2_ (μg/m^3^)	CO (mg/m^3^)
LP1	**N**	**N**	**N**	**N**	**−**	**+**
MP	**N**	**+**	**+**	**N**	**N**	**N**
QRF	**N**	**N**	**N**	**N**	**N**	**N**
CP	**N**	**N**	**N**	**N**	**N**	**N**
ADV	**N**	**N**	**N**	**−**	**+**	**N**
RSV	**N**	**N**	**N**	**N**	**−**	**N**
FluA	**N**	**N**	**N**	**N**	**N**	**N**
FluB	**+**	**+**	**+**	**+**	**+**	**N**
PIVs	**N**	**N**	**N**	**N**	**N**	**N**

### Comparison of prevalence of respiratory pathogens between low- and high-concentration groups of air pollutants

**Figure 4:**
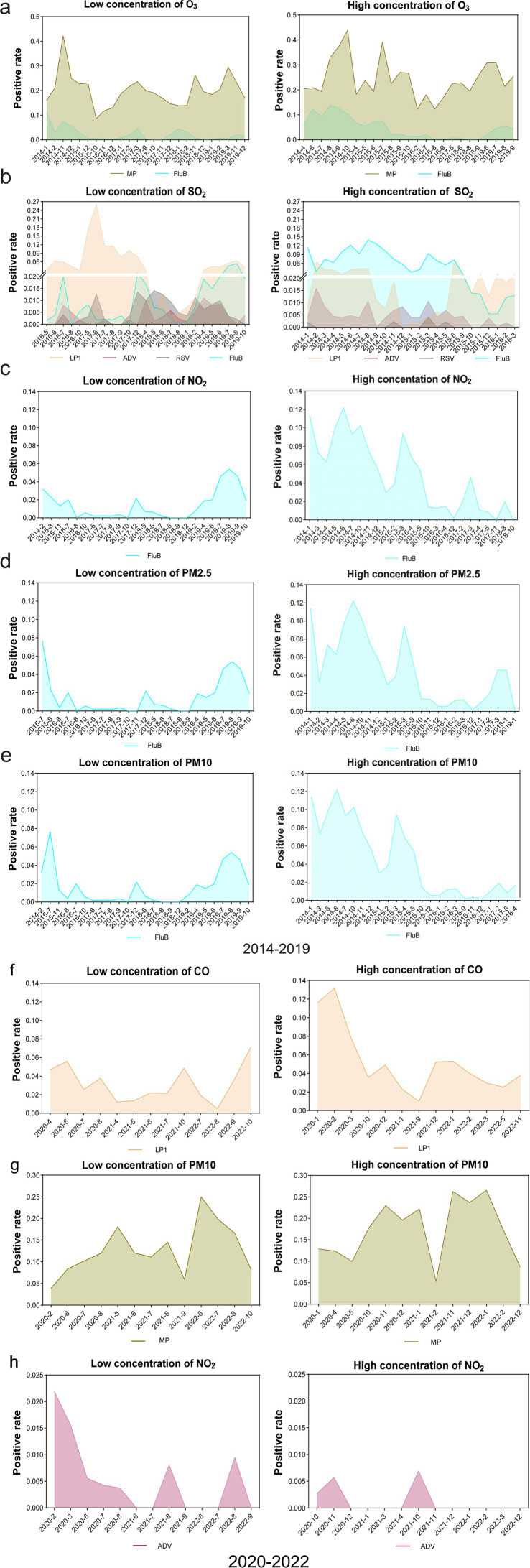
Comparison of the prevalence of respiratory pathogens between low- and high-concentration groups of air pollutants. **(A)** Comparison of the prevalence of MP and FluB in low- and high-concentration groups of O_3_, respectively, 2014–2019. **(B)** Comparison of the prevalence of LP1, ADV, RSV, and FluB in low- and high-concentration groups of SO_2_, respectively, 2014–2019._._ **(C–E)** Comparison of the prevalence of FluB in low and high concentration groups of NO_2_, PM2.5, and PM10, 2014–2019. **(F–H)** Comparison of the prevalence of LP1, MP, and ADV in low- and high-concentration groups of CO, PM10, and NO_2_, respectively, 2020–2022.

Building upon the significant linear correlation observed among the aforementioned groups, we conducted further analysis to determine the impact of low and high levels of air pollutants on the prevalence patterns of specific pathogens. The susceptibility of the affected population to the spread of respiratory pathogens varies depending on the concentration of different air pollutants. Our findings in the period from 2014 to 2019 indicated that elevated levels of O_3_ were associated with a significant increase in the prevalence of MP and FluB (Figure 4A), and heightened SO_2_ concentrations were strongly linked to the increase in the prevalence of FluB and ADV (Figure 4B). Except CO, which exhibited no influence on FluB prevalence, the other five air pollutants (O_3_, SO_2_, NO_2_, PM2.5, and PM10) with high concentrations contributed to an increased risk of FluB infection. In addition, high levels of NO_2_ (Figure 4C), PM2.5 (Figure 4D), and PM10 (Figure 4E) were found to elevate the positive rate of FluB significantly, while high levels of SO_2_ oppositely decreased the prevalence of LP1 and RSV (Figure 4B). However, our results in the period from 2020 to 2022 showed a big difference compared with the above results and suggested that increased CO levels were associated with a higher LP1 (Figure 4F) prevalence. Similarly, heightened PM10 concentrations positively correlated with an increase in the occurrence of MP (Figure 4G). Conversely, the elevated concentration of NO_2_ diminished the occurrence of ADV (Figure 4H).

## Discussion

Ambient air pollution is a high-risk factor for viral respiratory infections, including a possible mechanism of inflammation, cell death, oxidative stress, and expression of virus receptors (14). Our research suggested that the rise of air pollutants is associated with the prevalence of respiratory pathogens. This study, which is characterized by its extensive scale and the longest duration of follow-up, represents the most comprehensive investigation of the impact of ambient air pollution on population’s health in Wuhan City, Central China, with a particular focus on respiratory pathogen transmission and infection, to date.

According to the International Agency for Research on Cancer (IARC) of the World Health Organization (WHO), outdoor air pollution is classified as a Group 1 carcinogen. Atmospheric pollutants can be categorized into gaseous pollutants and total suspended particles (TSPs). The former encompasses substances such as CO, SO_2_, NO_2_, and O_3_, whereas the latter predominantly comprises PM2.5 and PM10 particles. It has been postulated that exposure to air pollutants can trigger the generation of reactive oxygen species (ROS), induce oxidative stress, and enhance mucus secretion and cytokines production, therefore exerting detrimental effects on pulmonary health (12, 15). Furthermore, air pollution has been shown to modulate host defense mechanisms. Research findings suggested that exposure to air pollutants might impair the phagocytic capacity of macrophages and disrupt immune function (16). The aforementioned factors collectively augment the population’s susceptibility to respiratory pathogen infections. Several references have confirmed some of our conclusions. In this study, we observed significant associations between air pollutants and the detection rates of FluB, ADV, MP, and LP1. The prevalence of FluB was found to increase in association with high levels of certain air pollutants. Studies have indicated that PM2.5, PM10, SO_2_, and CO can increase the risk of influenza virus infection (13, 17, 18). Moreover, a study in Singapore13 also showed a positive correlation between SO_2_, NO_2_, and the prevalence of FluB. Another study reported a negative relationship between CO and RTIs which was contrary to our findings (19). Studies have shown that SO_2_ is a well-known inducer of inflammation in the respiratory system and leads to a direct damage to the airways and destruction of respiratory barrier function (20–22). NO_2_, a primary pollutant emitted from traffic and a major constituent of urban air pollution, predominantly affects deep bronchioles and alveoli in the respiratory system and results in a strong irritation to lung tissues. The mechanisms underlying NO_2_-induced damage to the respiratory system are primarily characterized by inflammatory reactions (23), oxidative stress (24), and increased airway reactivity (25). Previous studies have found that long-term inhalation of O_3_ was also more likely to induce or aggravate the respiratory system disease (26), and another study also shows that the extremely high concentrations of O_3_ play a promoting role in the pathogenesis of influenza as well (18, 27). This may elucidate the positive association between O_3_ and FluB infections observed in our study as high O_3_ concentrations can concomitantly increase the susceptibility to FluB infection. Inhalation of high concentrations of O_3_ can trigger the influx of neutrophils into the airways, leading to plasma protein loss and lung permeability reduction, which causes acute injury to the airways and weakens lung function (28). Meanwhile, we first discovered a positive correlation between SO_2_ and ADV infection, indicating that high levels of SO_2_ can enhance ADV infection. This conclusion has not been previously reported both in domestic or international studies. Our research demonstrated a predominant occurrence of MP infection among children aged 1–7 years, with the detection rate exhibiting a positive correlation with O_3_ concentration. A study in Suzhou revealed that there was a positive and statistically significant association between O_3_ and MP infection in children under 6 years of age (*p* < 0.05), with the largest effect sizes at a lag of 4 weeks (7), aligning with the previous report and our own findings.

To reduce the spread of the sudden outbreak of SARS-CoV-2 at the end of 2019 after 2020, a series of non-pharmaceutical interventions were implemented, such as reducing personnel travel and mobility and limiting the use of private cars and public transportation. These measures can reduce the emission of toxic and harmful gases in the atmosphere and significantly change the air pollution situation and epidemiological characteristics of respiratory pathogens in the Wuhan region (29). Consequently, we analyzed the air pollution data and prevalence of respiratory pathogens for 2014–2019 and 2020–2022 separately. The obvious disparities in outcomes between these two time periods may be attributed to the changes in the concentration of different air pollutants, which have the potential to influence and alter the prevalence of diverse respiratory pathogens. Our study has shown that the overall air pollution from 2020 to 2022 was better than 2014–2019, and there have been dramatic changes in the correlation between different air pollutants and specific respiratory pathogens. A finding in Chengdu has proved that there was a negative correlation between the positive rate of MP and PM2.5 as well as PM10 among children (30). This is completely opposite to the conclusion we have obtained. Nevertheless, another study has indicated a significant association between MP infection and PM10 exposure in pediatric populations (7). It has been demonstrated that there is a significant association between the average concentration of CO and hospitalization rates among patients with respiratory system diseases, with an RR of 1.045 (95% *CI*: 1.009–1.082) (31). However, current evidence does not provide clear proof that hospitalization caused by respiratory diseases is due to LP1. Nevertheless, our study has revealed a significant positive correlation between CO and LP1. Some of our conclusions (for example, we first discovered the positive correlation between SO_2_ and ADV infection and between CO and LP1 infection.) have not been reported in previous studies yet, but there were references (20–22, 31) indicating that SO_2_ and CO do increase the risk of respiratory tract infections. Thus, our conclusions will serve as a predictor and play a bridging role in subsequent related research. The relationship between air pollutants and susceptibility to respiratory pathogens varies across different countries and regions and exhibits inconsistent effects of air pollution on the prevalence of respiratory pathogens. It may be potentially resulted in differences in lifestyle, levels and composition of air pollution, weather conditions, and local demographic characteristics, such as age distribution of survey participants, gender, and education level.

## Conclusion

In summary, this study not only unveils the temporal patterns of prevalence changes in nine respiratory pathogens from 2014 to 2022, but more importantly, it elucidates the intricate association between ambient air pollutants and respiratory pathogens. The General Hospital of Central Theater Command exhibited the highest infectivity of MP among nine tested respiratory pathogens from 2014 to 2022; an elevated level of O_3_ has been confirmed to increase the incidence of MP infection. Some of our conclusions are consistent with the previous studies, and we are also presenting new findings for the first time. We first discovered a positive correlation between SO_2_ and ADV infection, indicating that high levels of SO_2_ can enhance ADV prevalence. Our study also revealed a significant positive correlation between CO and LP1, while no direct evidence was provided to prove the relationship between them in previous research studies. Moreover, we have innovatively discovered that high levels of air pollutants (PM2.5, PM10, O_3_, NO_2_, SO_2_, and CO) can indeed contribute to an elevated prevalence of specific respiratory pathogens (MP, FluB, ADV, and LP1) compared to low pollution groups that have not been reported before, emphasizing the urgent need for effective control measures targeting air pollution to mitigate the spread of respiratory pathogens in future.

## Data Availability

The original contributions presented in the study are included in the article/Supplementary material, further inquiries can be directed to the corresponding authors.
